# Development and Protective Evaluation of an O18–CRM197 Glycoconjugate Candidate Preparation Against APEC O18 Challenge in SPF Chickens

**DOI:** 10.3390/microorganisms14092082

**Published:** 2026-09-17

**Authors:** Tingfan Zhu, Jing Yuan, Kangmei Feng, Hairui Bai, Caihong Yan, Yang Yang, Aijian Qin, Wenjie Jin

**Affiliations:** 1 College of Veterinary Medicine, Yangzhou University, Yangzhou 225009, China; zhutingfan@126.com (T.Z.); thissurjing@163.com (J.Y.); fengkangmei@163.com (K.F.); bi2881110333332@163.com (H.B.); yancaihong999@163.com (C.Y.); yy@yzu.edu.cn (Y.Y.); aijian@yzu.edu.cn (A.Q.); 2 Jiangsu Co-Innovation Center for Prevention and Control of Important Animal Infectious Diseases and Zoonosis, Yangzhou 225009, China; 3 Animal Disease Testing and Technical Service Center, Yangzhou University, Yangzhou 225009, China; 4 Key Laboratory of Jiangsu Preventive Veterinary Medicine, Ministry of Education, Yangzhou 225009, China

**Keywords:** avian pathogenic *Escherichia coli*, APEC O18, O18–CRM197, glycoconjugate vaccine, SPF chickens, immune protection

## Abstract

Avian pathogenic *Escherichia coli* (APEC) is an important cause of colibacillosis in poultry, and increasing antimicrobial resistance has highlighted the need for alternative preventive strategies. This study aimed to develop an O18-cross-reacting material 197 (O18–CRM197) glycoconjugate vaccine candidate using an *Escherichia coli*-based protein glycan coupling technology (PGCT) platform and to evaluate its immunogenicity and preliminary protective performance in specific-pathogen-free (SPF) chickens. A CLM24-derived production host, Y03, was generated by sequential deletion of *lpxM*, *zwf*, and *gldA* using a CRISPR/Cas9- and λ-Red-mediated genome editing system. An O18 O-polysaccharide expression plasmid and a co-expression plasmid encoding PglB and a modified CRM197 carrier protein were introduced into Y03 for in vivo glycoconjugate production. Western blotting showed high-molecular-weight signals recognized by both anti-CRM197 antibody and anti-O18 serum, supporting production of an O18–CRM197 glycoconjugate candidate. Immunization with the adjuvanted purified candidate induced O18-specific serum immunoglobulin G (IgG) responses in SPF chickens. After homologous APEC O18 challenge at 1 × 10^9^ colony-forming units (CFU)/bird, immunized chickens showed reduced gross lesion scores and bacterial burdens in blood and organs, with the 50 μg dose showing more consistent protective performance than the 30 μg dose. These findings provide preliminary evidence that the adjuvanted purified O18–CRM197 candidate preparation has immunogenicity and protective potential against homologous APEC O18 challenge.

## 1. Introduction

Avian pathogenic *Escherichia coli* (APEC) is one of the most important bacterial pathogens affecting poultry production and a major cause of colibacillosis in chickens [1,2]. Infection with APEC can lead to septicemia, pericarditis, airsacculitis, perihepatitis, salpingitis, reduced growth performance, and increased mortality, thereby causing substantial economic losses in the poultry industry [3]. In recent years, the extensive use of antimicrobials in poultry production has contributed to the emergence of multidrug-resistant APEC strains, making prevention and control increasingly difficult. With the ongoing reduction in antibiotic use in food-animal production, the development of safe and effective vaccines has become an important strategy for improving flock health and reducing antibiotic dependence [4].

Among the pathogenic serotypes of APEC, O18 is considered one of the important circulating serotypes [5,6]. The O-polysaccharide (O-PS) component of lipopolysaccharide (LPS) is a major surface antigen and an attractive vaccine target because of its serotype specificity and capacity to induce protective humoral immunity [7,8]. However, polysaccharides alone are usually T cell-independent antigens and often fail to induce strong, long-lasting immune responses, especially in young animals [9,10]. The conjugation of polysaccharides to a carrier protein can convert them into T cell-dependent antigens, thereby enhancing immunogenicity and promoting immunological memory [11,12].

Protein glycan coupling technology (PGCT) offers an alternative to conventional chemical conjugation by enabling covalent linkage of polysaccharides to carrier proteins directly in engineered *E. coli* [13,14]. Compared to in vitro chemical conjugation, this strategy can simplify production, reduce batch-to-batch variation, and preserve native glycan epitopes [15]. In the present study, we sought to develop an O18–CRM197 glycoconjugate vaccine candidate against APEC by establishing an *E. coli*-based N-glycosylation system that couples O18 O-polysaccharide to the carrier protein CRM197. To achieve this objective, we constructed a CLM24-derived engineered production host, established the glycoengineering platform for in vivo glycoconjugate production, and evaluated the immunogenicity and preliminary protective efficacy of the purified candidate in specific-pathogen-free (SPF) chickens.

## 2. Materials and Methods

### 2.1. Bacterial Strains, Plasmids, and Antibodies

The bacterial strains, plasmids, primers, antibodies, and experimental animals used in this study are summarized in Table 1 and Supplementary Table S1. *Escherichia coli* W3110 was used as the original reference strain. The previously established PGCT-compatible chassis strain CLM24, a W3110-derived Δ*waaL* strain, was used as the parental strain for construction of the engineered production host Y03. *E. coli* DH5α was used for routine plasmid construction and propagation.

The homologous APEC O18 strain was used as both the source of the O18 O-polysaccharide biosynthesis gene cluster and the challenge strain in the animal experiment. The O18 O-polysaccharide expression plasmid pET-32a(+)-O18 was constructed in this study. The PglB–CRM197 co-expression plasmid pCRM197-pglB was generated based on the previously established pCRM-4 plasmid and was used to express PglB and the modified CRM197 carrier protein. Rabbit anti-O18 serum and anti-CRM197 antibody were used for Western blot analysis. Horseradish peroxidase (HRP)-conjugated secondary antibodies were used for immunoblot detection, and HRP-conjugated goat anti-chicken IgG was used for enzyme-linked immunosorbent assay (ELISA) detection of chicken serum IgG.

Seven-day-old SPF chickens were used for immunization and homologous challenge experiments. The sex of the chickens was not determined. Birds were housed in SPF animal facilities under standard husbandry conditions, with free access to feed and water. Chickens were randomly assigned to experimental groups before immunization. Clinical monitoring was conducted at least once daily after challenge. Predefined humane endpoints included moribund status, persistent recumbency, inability to access feed or water, severe respiratory distress, severe or persistent bloody diarrhea, or any condition judged to cause excessive suffering. Birds reaching humane endpoints were humanely euthanized immediately and recorded as deaths for outcome analysis. At scheduled sampling time points or at the end of the observation period, all remaining birds were humanely euthanized under anesthesia with 4–5% isoflurane until loss of consciousness, followed by intraperitoneal injection of sodium pentobarbital at 100 mg/kg body weight. Carcasses were transferred to the Animal Remains Harmless Disposal Center of Yangzhou University for processing according to institutional regulations. Gross lesion scoring was performed according to predefined criteria. Allocation concealment and blinded outcome assessment were not formally performed. The animal experiment protocol was approved by the Institutional Animal Care and Use Committee of Yangzhou University under approval number 202603399. All procedures were performed in accordance with relevant guidelines and regulations and adhered to the Animal Research: Reporting of In Vivo Experiments (ARRIVE) guidelines.

### 2.2. Construction of Y03 and Assessment of Bacterial Growth

The engineered production host Y03 was constructed from the previously established PGCT-compatible chassis strain CLM24. CLM24 is a W3110-derived Δ*waaL* strain and was used as the parental strain for further genome engineering. To generate Y03, *lpxM*, *zwf*, and *gldA* were sequentially deleted from the CLM24 background using a CRISPR/Cas9- and λ-Red-mediated dual-plasmid genome editing system.

Briefly, the helper plasmid pHCY-25A, carrying Cas9, the λ-Red recombination system, and the sacB counter-selection marker, was introduced into CLM24. Gene-specific pHCY-26D-editing plasmids containing sgRNA cassettes and homologous repair arms were constructed for deletion of *lpxM*, *zwf*, and *gldA*. Genome editing was induced by isopropyl β-D-1-thiogalactopyranoside (IPTG) and L- (+)-arabinose according to the editing system protocol. After each editing step, candidate colonies were screened by colony polymerase chain reaction (PCR) using primers flanking the corresponding target locus.

The *lpxM* gene was first deleted from CLM24 to generate Y01, with positive clones identified by the expected amplicon shift from 2617 bp to 1645 bp. Subsequently, *zwf* was deleted from Y01 to generate Y02, as confirmed by the expected shift from 2584 bp to 1278 bp. Finally, *gldA* was deleted from Y02 to generate Y03, with positive clones confirmed by the expected shift from 2260 bp to 1100 bp. After each deletion step, the temperature-sensitive pHCY-26D editing plasmid was cured before the next round of editing. The helper plasmid pHCY-25A was removed by sucrose counter-selection after completion of genome editing. The final engineered strain Y03 was defined as *E. coli* W3110 Δ*waaL* Δ*lpxM* Δ*zwf* Δ*gldA*. All primers used for plasmid construction and PCR verification are listed in Supplementary Table S1.

To evaluate whether sequential deletion of *lpxM*, *zwf*, and *gldA* affected bacterial growth, W3110, CLM24, Y01, Y02, and Y03 were cultured overnight in Luria–Bertani (LB) broth at 37 °C with shaking at 225 rpm. The overnight cultures were diluted 1:100 into fresh LB broth and incubated at 37 °C with shaking. For each strain, 200 μL of culture was transferred into a 96-well plate, and optical density at 600 nm (OD600) values were measured every 2 h for 12 h using a multifunctional microplate reader (BioTek Instruments, Winooski, VT, USA).

### 2.3. Construction and Verification of pET-32a(+)-O18

The O18 O-polysaccharide biosynthesis gene cluster was amplified from the genomic DNA of a homologous APEC O18. Because the full-length O18 O-polysaccharide gene cluster is approximately 12 kb, it was amplified as two fragments, O18-1 and O18-2, using primer pairs O18-1F/O18-1R and O18-2F/O18-2R, respectively. The pET-32a(+) vector was linearized by PCR using primers F03/F04. PCR products of the expected sizes were purified using a gel extraction kit.

The purified O18-1 fragment, O18-2 fragment, and linearized pET-32a(+) vector were assembled using a One Step Cloning Kit according to the manufacturer’s instructions. The recombinant plasmid was transformed into *E. coli* DH5α competent cells, and positive clones were screened by colony PCR using primers 443/F07. The confirmed plasmid was designated pET-32a(+)-O18.

To verify functional expression of the O18 O-polysaccharide module, pET-32a(+)-O18 was transformed into *E. coli* W3110 to generate W3110/pET-32a(+)-O18. Positive transformants were confirmed by colony PCR using primers F05/O18-3R. W3110/pET-32a(+)-O18 was cultured in 2 × YT broth and induced with IPTG. Homologous APEC O18 and W3110 were used as positive and negative controls, respectively. Lipopolysaccharide samples were extracted using an LPS Extraction Kit according to the manufacturer’s instructions.

For Western blot analysis, extracted LPS samples were separated by sodium dodecyl sulfate–polyacrylamide gel electrophoresis (SDS-PAGE) and transferred onto nitrocellulose membranes. Membranes were blocked with 5% skim milk in phosphate-buffered saline containing Tween-20 (PBST) and incubated with rabbit anti-O18 serum diluted 1:400, followed by HRP-conjugated goat anti-rabbit IgG diluted 1:30,000. Signals were developed using enhanced chemiluminescence reagent and visualized with an imaging system. Detection of O18-reactive ladder-like bands in W3110/pET-32a(+)-O18 was used to verify functional expression of the O18 O-polysaccharide module.

### 2.4. Construction and Verification of pCRM197-pglB

The PglB–CRM197 co-expression plasmid pCRM197-pglB was constructed based on the previously established pCRM-4 plasmid. This plasmid encodes the oligosaccharyltransferase PglB and a modified CRM197 carrier protein containing a signal peptide, a PglB-recognized glycosylation acceptor sequence, and a His tag. The signal peptide was included to direct CRM197 to the appropriate cellular compartment for PglB-mediated glycan transfer, whereas the His tag was used for downstream affinity purification. To improve compatibility with the O18 O-polysaccharide expression plasmid pET-32a(+)-O18, the ColE1-derived replicon of pCRM-4 was replaced with a p15A replicon. The resulting plasmid was designated pCRM197-pglB.

To verify expression of the CRM197-containing module, pCRM197-pglB was transformed into the engineered host strain Y03. Positive transformants were identified by colony PCR and designated Y03/pCRM197-pglB. After induction, whole-cell protein samples were prepared and analyzed by Western blot using anti-CRM197 antibody. Non-transformed Y03 was used as the negative control. Detection of an approximately 65 kDa band in Y03/pCRM197-pglB, but not in Y03, was used to verify expression of the CRM197-containing module.

### 2.5. Production, Purification, and Characterization of the O18–CRM197 Glycoconjugate Candidate

To produce the O18–CRM197 glycoconjugate candidate, pET-32a(+)-O18 was introduced into Y03/pCRM197-pglB by electroporation, generating the recombinant production strain Y03/pCRM197-pglB/pET-32a(+)-O18. Positive transformants were selected on LB agar plates containing the corresponding antibiotics at 50 μg/mL and confirmed by colony PCR.

A confirmed recombinant clone was cultured overnight in LB broth containing the corresponding antibiotics at 50 μg/mL and then diluted into fresh terrific broth (TB) medium for glycoconjugate production. When the culture reached an OD600 of 0.6–0.8, expression of the PglB–CRM197 module was induced with IPTG, followed by incubation at 16 °C overnight for glycoconjugate production. After induction, bacterial cells were harvested, washed with phosphate-buffered saline (PBS), resuspended in lysis buffer, and disrupted by sonication on ice. The lysate was centrifuged to remove cell debris, and the clarified supernatant was used for purification.

Because the CRM197-containing construct carried a His-tag, the O18–CRM197 glycoconjugate candidate was purified by nickel-nitrilotriacetic acid (Ni-NTA) affinity chromatography. The bound product was eluted with imidazole-containing buffer, and eluted fractions containing the target product were dialyzed against PBS. The purified preparation was stored at −80 °C until further analysis and animal immunization. Protein concentration was determined using a BCA assay with bovine serum albumin as the standard, and total carbohydrate content was measured by the phenol–sulfuric acid method with glucose as the standard.

Western blot analysis was performed to characterize the purified candidate. For anti-CRM197 detection, purified products from Y03/pCRM197-pglB and Y03/pCRM197-pglB/pET-32a(+)-O18 were analyzed, with the product from Y03/pCRM197-pglB used as the unglycosylated CRM197 control. For anti-O18 detection, homologous APEC O18 LPS and W3110 LPS were used as positive and negative controls, respectively. Samples were separated by SDS-PAGE, transferred onto nitrocellulose membranes, and blocked with 5% skim milk in PBST. Membranes were incubated with anti-CRM197 antibody or rabbit anti-O18 serum, followed by the corresponding HRP-conjugated secondary antibodies. Signals were developed using enhanced chemiluminescence reagent and visualized with an imaging system. High-molecular-weight ladder-like signals detected by both anti-CRM197 antibody and anti-O18 serum were considered supportive evidence for production of the O18–CRM197 glycoconjugate candidate.

Residual endotoxin levels in the purified O18–CRM197 glycoconjugate candidate preparation were further quantified using a recombinant Factor C (rFC)-based endotoxin detection kit (MedChemExpress, MCE, Monmouth Junction, NJ, USA). Briefly, appropriately diluted purified samples were incubated with the rFC reaction mixture, and fluorescence intensity was measured at excitation/emission wavelengths of 380/440 nm after 1 h incubation. Endotoxin concentrations were calculated according to a standard curve generated using endotoxin standards ranging from 0.005 to 5 EU/mL.

### 2.6. Immunization, Homologous Challenge, and Protective Efficacy Evaluation

Sixty 7-day-old SPF chickens were randomly divided into four groups, with 15 birds per group: a 30 μg O18–CRM197 immunization group, a 50 μg O18–CRM197 immunization group, a challenge control group, and a blank control group. The immunization dose was calculated based on the protein concentration of the purified candidate preparation. In each group, five birds were assigned for bacterial burden analysis at 24 h post-challenge, eight birds were used for clinical observation, body weight measurement, serum antibody detection, and gross lesion scoring, and two backup birds were not included in statistical analyses.

Chickens in the immunization groups were vaccinated subcutaneously in the neck with purified O18–CRM197 glycoconjugate candidate at 30 μg/bird or 50 μg/bird. Before immunization, the purified O18–CRM197 glycoconjugate candidate was emulsified with a laboratory-prepared oil-based emulsion adjuvant consisting of 90% (*v*/*v*) white oil, 6% (*v*/*v*) Span 80, and 4% (*v*/*v*) Tween 80, at an antigen solution-to-adjuvant ratio of 3:1 (*v*/*v*). Chickens in the challenge control and blank control groups received 0.2 mL PBS by the same route. A booster immunization was administered two weeks later using the same dose and route.

Two weeks after booster immunization, chickens in the two immunization groups and the challenge control group were challenged subcutaneously in the neck with homologous APEC O18 at 1 × 10^9^ CFU/bird in 0.2 mL. This challenge dose was selected based on preliminary pathogenicity assessment in SPF chickens. The blank control group received an equal volume of PBS. Clinical signs and mortality were recorded daily for 10 days after challenge.

Body weight was recorded before challenge and at 10 days post-challenge. Gross lesions of the air sacs, heart, and liver were scored from 0 to 3 for each organ, and the total gross lesion score was calculated with a maximum score of 9 per bird. Chickens that died during the observation period were assigned the maximum lesion score.

O18-specific serum IgG was measured by indirect ELISA. Serum samples were collected from four birds per group every 7 days after immunization and at 10 days post-challenge. *E. coli* O18 LPS was used as the coating antigen at 0.25 μg/mL. Serum samples were diluted 1:200, and HRP-conjugated goat anti-chicken IgG was used as the secondary antibody at 1:200. Absorbance was measured at 450 nm.

For bacterial burden analysis, five birds from each challenged group were euthanized at 24 h post-challenge. Heart blood, liver, spleen, and lung samples were collected aseptically. Tissue samples were homogenized in sterile PBS, serially diluted, and plated onto LB agar using the drop-plate method. After incubation at 37 °C for 16 h, colonies were counted, and bacterial burdens were calculated as CFU/mL for heart blood and CFU/g for tissues.

### 2.7. Statistical Analysis

Data were analyzed using GraphPad Prism 10. Normality was assessed using the Shapiro–Wilk test, and homogeneity of variance was assessed using Levene’s test or the Brown–Forsythe test. Bacterial burden data were log10-transformed before analysis. Growth curves and serum IgG responses were analyzed using two-way analysis of variance (ANOVA) followed by multiple-comparison tests. Body weight gain was analyzed using one-way ANOVA followed by Tukey’s multiple-comparison test. Gross lesion scores were analyzed using the Mann–Whitney U test or Kruskal–Wallis test followed by Dunn’s multiple-comparison test where appropriate. Bacterial burdens were compared between each immunized group and the challenge control group using one-way ANOVA followed by Dunnett’s test or non-parametric tests when assumptions were not met. Differences were considered statistically significant at *p* < 0.05.

## 3. Results

### 3.1. Construction and Growth Characterization of Y03

To generate an engineered host for O18–CRM197 glycoconjugate production, the previously established PGCT-compatible chassis strain CLM24 was further modified by sequential deletion of *lpxM*, *zwf*, and *gldA*. Candidate colonies were screened by PCR after each editing step, and confirmed intermediate strains were used for subsequent editing.

**Figure 1:**
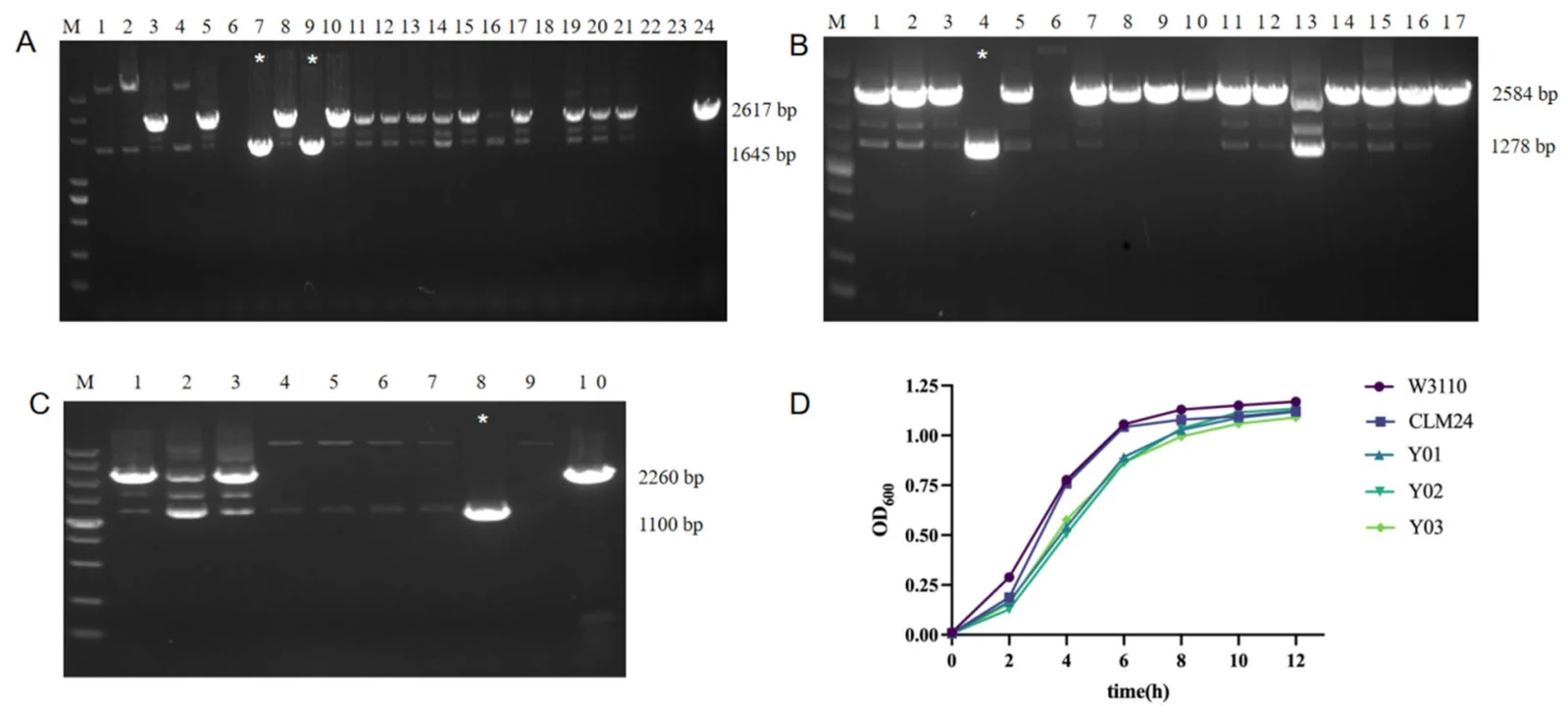
Construction and growth characterization of the Y03 production host. (**A**) PCR verification of *lpxM* deletion from CLM24. Lane M, DL5000 DNA marker; lanes 1–23, candidate clones; lane 24, parental CLM24. Lanes 7 and 9, indicated by asterisks, showed single PCR products corresponding to the expected deletion size of 1645 bp and were identified as positive *lpxM* deletion clones, whereas the parental CLM24 strain produced a 2617 bp amplicon. (**B**) PCR verification of *zwf* deletion from Y01. Lane M, DL5000 DNA marker; lanes 1–16, candidate clones; lane 17, parental Y01. Lane 4, indicated by an asterisk, showed the expected 1278 bp deletion product and was identified as a positive *zwf* deletion clone, whereas the parental Y01 strain produced a 2584 bp amplicon. (**C**) PCR verification of *gldA* deletion from Y02. Lane M, DL5000 DNA marker; lanes 1–9, candidate clones; lane 10, parental Y02. Lane 8, indicated by an asterisk, showed the expected 1100 bp deletion product and was identified as a positive *gldA* deletion clone, whereas the parental Y02 strain produced a 2260 bp amplicon. (**D**) Growth curves of W3110, CLM24, and CLM24-derived engineered strains. W3110, CLM24, Y01, Y02, and Y03 were cultured in LB broth at 37 °C with shaking, and OD600 values were measured every 2 h for 12 h. No significant differences in growth were observed among the tested strains under these conditions (*p* > 0.05).

Deletion of lpxM from CLM24 generated Y01. PCR screening showed that candidate clones in lanes 7 and 9 produced single bands of the expected deletion size of 1645 bp, whereas the parental CLM24 strain produced a 2617 bp amplicon (Figure 1A). Subsequently, *zwf* was deleted from Y01 to generate Y02, with the positive clone in lane 4 showing the expected 1278 bp deletion product instead of the 2584 bp parental amplicon (Figure 1B). Finally, *gldA* was deleted from Y02 to generate Y03, and the positive clone in lane 8 produced the expected 1100 bp deletion product, whereas the parental Y02 strain produced a 2260 bp amplicon (Figure 1C). These results confirmed the successful sequential deletion of *lpxM*, *zwf*, and *gldA* from the CLM24 background. The final genotype of Y03 was defined as *E. coli* W3110 Δ*waaL* Δ*lpxM* Δ*zwf* Δ*gldA*.

Because Y03 was designed as the production host for glycoconjugate expression, we next evaluated whether the sequential genome modifications affected bacterial growth. W3110, CLM24, Y01, Y02, and Y03 showed similar growth patterns during 12 h of cultivation in LB broth, and no significant differences in overall growth were observed among the tested strains under these conditions (*p* > 0.05; Figure 1D). These results indicate that deletion of *lpxM*, *zwf*, and *gldA* did not cause an obvious growth defect in the CLM24-derived host, supporting the use of Y03 for subsequent O18–CRM197 glycoconjugate expression experiments.

### 3.2. Functional Verification of the O18 O-Polysaccharide and CRM197 Expression Modules

**Figure 2:**
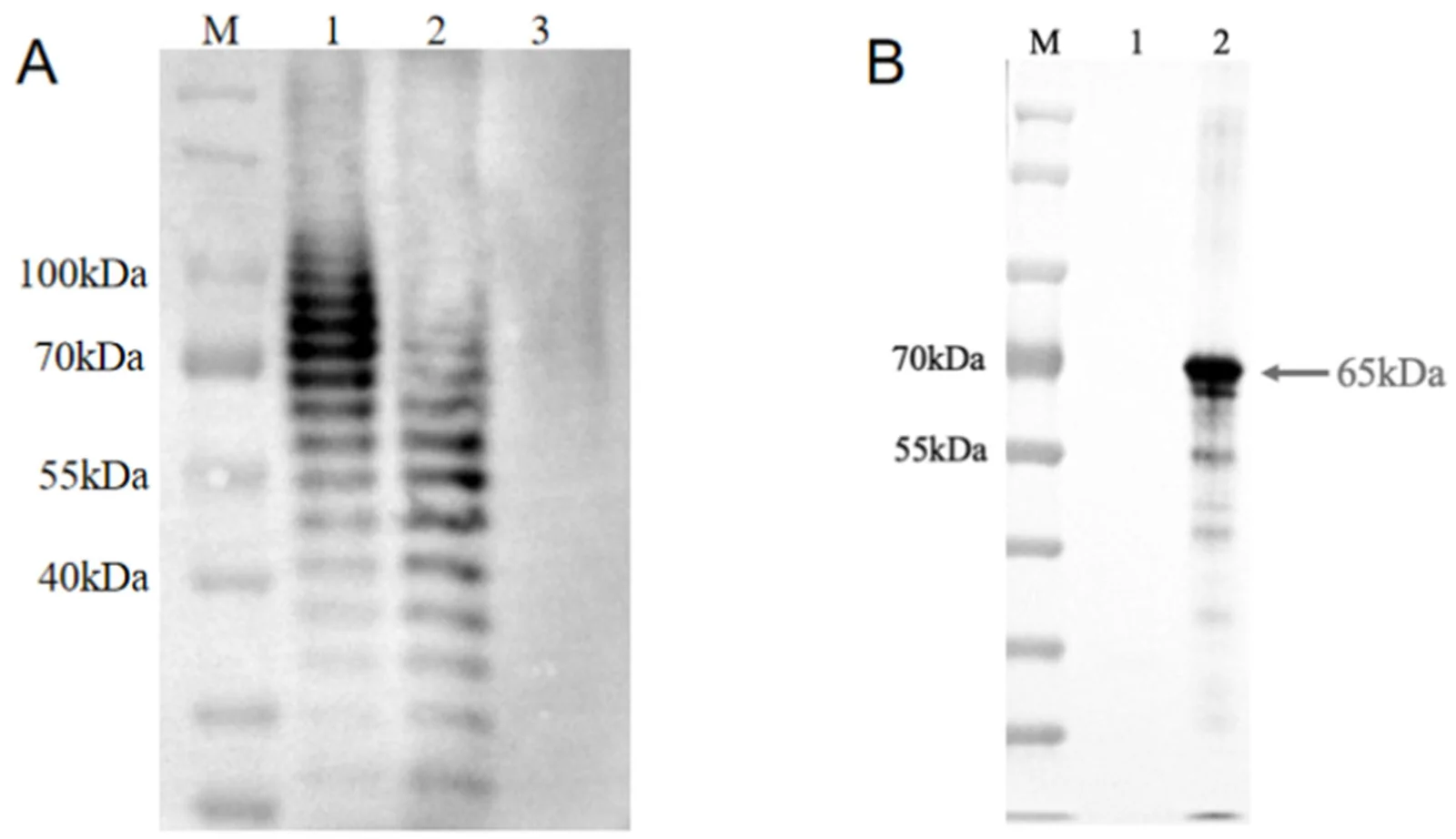
Functional verification of the O18 O-polysaccharide and CRM197 expression modules. (**A**) Western blot verification of O18 O-polysaccharide expression from pET-32a(+)-O18 using anti-O18 serum. LPS samples extracted from homologous APEC O18, W3110/pET-32a(+)-O18, and W3110 were analyzed. Lane M, protein marker; lane 1, homologous APEC O18 LPS; lane 2, W3110/pET-32a(+)-O18 LPS; lane 3, W3110 LPS. W3110/pET-32a(+)-O18 produced O18-reactive ladder-like signals similar to those of homologous APEC O18, whereas no corresponding signal was detected in W3110. (**B**) Western blot verification of CRM197 expression from pCRM197-pglB in the Y03 host using anti-CRM197 antibody. Whole-cell lysates from Y03 and Y03/pCRM197-pglB were analyzed. Lane M, protein marker; lane 1, Y03 control; lane 2, Y03/pCRM197-pglB. A distinct band of approximately 65 kDa was detected in Y03/pCRM197-pglB, whereas no corresponding signal was observed in the Y03 control.

To establish the O18–CRM197 glycoconjugate production system, the O18 O-polysaccharide module and the CRM197-containing module were first functionally verified. The O18 O-polysaccharide expression plasmid pET-32a(+)-O18 was transformed into *E. coli* W3110, and LPS samples were analyzed by Western blot using anti-O18 serum. Homologous APEC O18 LPS showed a typical ladder-like signal pattern, consistent with the presence of heterogeneous O-polysaccharide chains (Figure 2A). Similar O18-reactive ladder-like signals were detected in W3110/pET-32a(+)-O18, whereas no corresponding O18-specific signal was observed in the W3110 control. These results indicated that the O18 O-polysaccharide module carried by pET-32a(+)-O18 was functionally expressed in the W3110 background.

The CRM197-containing module was then evaluated in the Y03 host. The pCRM197-pglB plasmid, which encodes PglB and a modified CRM197 carrier protein, was introduced into Y03, and whole-cell lysates were analyzed by Western blot using anti-CRM197 antibody. A distinct band of approximately 65 kDa was detected in Y03/pCRM197-pglB, whereas no corresponding signal was observed in the non-transformed Y03 control (Figure 2B). These results confirmed expression of the CRM197-containing module in Y03.

Together, the successful detection of O18-reactive polysaccharide signals and CRM197-specific protein expression demonstrated that both modules required for O18–CRM197 glycoconjugate production were functional, supporting their use in the subsequent glycoconjugate expression system.

### 3.3. Production and Characterization of the O18–CRM197 Glycoconjugate Candidate

After functional verification of the O18 O-polysaccharide module and the CRM197-containing module, both plasmids were introduced into the engineered Y03 host to produce the O18–CRM197 glycoconjugate candidate. The recombinant strain Y03/pCRM197-pglB/pET-32a(+)-O18 was induced, and the target product was purified by nickel affinity chromatography for further characterization.

**Figure 3:**
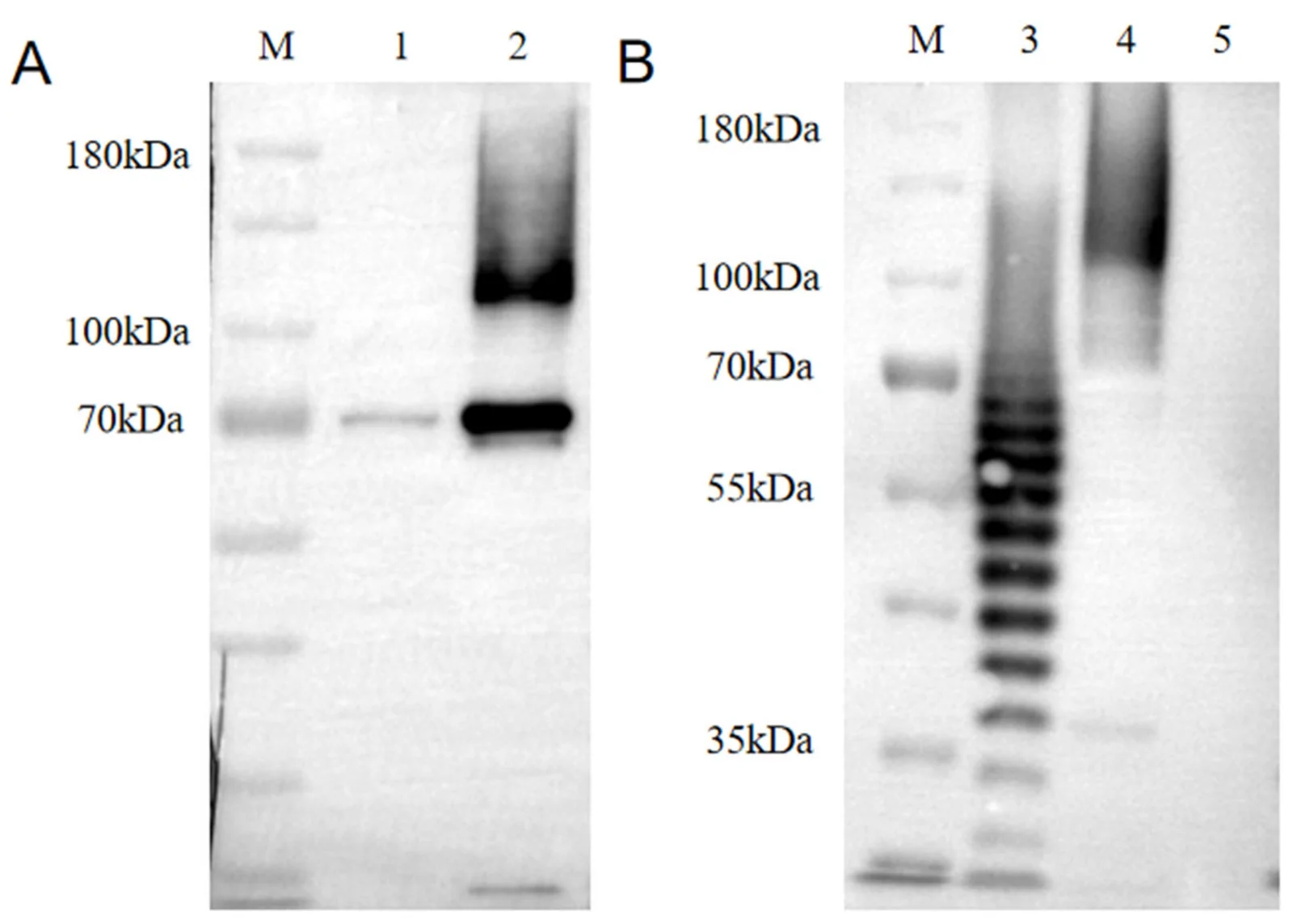
Western blot characterization of the purified O18–CRM197 glycoconjugate candidate. (**A**) Western blot analysis using anti-CRM197 antibody. Lane M, protein marker; lane 1, purified product from Y03/pCRM197-pglB; lane 2, purified product from Y03/pCRM197-pglB/pET-32a(+)-O18. High-molecular-weight ladder-like signals were detected in the product from the strain carrying both the O18 O-polysaccharide module and the CRM197-containing module. (**B**) Western blot analysis using anti-O18 serum. Lane M, protein marker; lane 3, homologous APEC O18 LPS; lane 4, purified product from Y03/pCRM197-pglB/pET-32a(+)-O18; lane 5, W3110 LPS. The purified product showed shifted high-molecular-weight O18-reactive signals, supporting production of the O18–CRM197 glycoconjugate candidate.

Western blot analysis using anti-CRM197 antibody showed that the control sample derived from Y03/pCRM197-pglB produced a major band corresponding to the unglycosylated CRM197 carrier protein. In contrast, the purified product from Y03/pCRM197-pglB/pET-32a(+)-O18 displayed additional high-molecular-weight ladder-like signals above 100 kDa (Figure 3A). The appearance of these higher-molecular-weight ladder-like signals suggested the generation of CRM197-associated glycan-modified products in the O18 glycoengineering system.

The purified product was further analyzed using anti-O18 serum. Homologous APEC O18 LPS showed the expected ladder-like signal pattern, whereas the purified product displayed shifted high-molecular-weight ladder-like signals that were also recognized by anti-O18 serum (Figure 3B). The detection of high-molecular-weight signals recognized by both anti-CRM197 and anti-O18 antibodies provided supportive evidence for the production of an O18–CRM197 glycoconjugate candidate in the Y03 host.

The purified preparation was also analyzed for protein and carbohydrate contents. The protein concentration was 258.68 μg/mL, and the total carbohydrate concentration was 132.94 μg/mL, corresponding to a carbohydrate-to-protein mass ratio of approximately 0.51. These results indicated that the purified candidate contained both protein and carbohydrate components, providing additional compositional evidence supporting the recovery of the O18–CRM197 glycoconjugate candidate preparation. The endotoxin standard curve showed excellent linearity within the tested range (R^2^ = 0.9981). The residual endotoxin level in the purified O18–CRM197 candidate preparation was approximately 10 EU/mL, corresponding to 38.66 EU/mg protein. Considering the immunization dose used in this study, the amount of residual endotoxin administered per chicken was further calculated. For the 30 μg and 50 μg immunization groups, the corresponding endotoxin exposure was approximately 1.16 EU/bird and 1.93 EU/bird, respectively.

### 3.4. O18–CRM197 Immunization Induced Humoral Responses and Improved Protective Outcomes After Homologous APEC O18 Challenge

To evaluate the immunogenicity and protective performance of the purified O18–CRM197 candidate preparation, SPF chickens were immunized with 30 μg or 50 μg of the adjuvanted purified candidate and subsequently challenged with homologous APEC O18. Serum IgG responses, body weight gain, clinical signs, gross lesion scores, and bacterial burdens were assessed after immunization and challenge.

**Figure 4:**
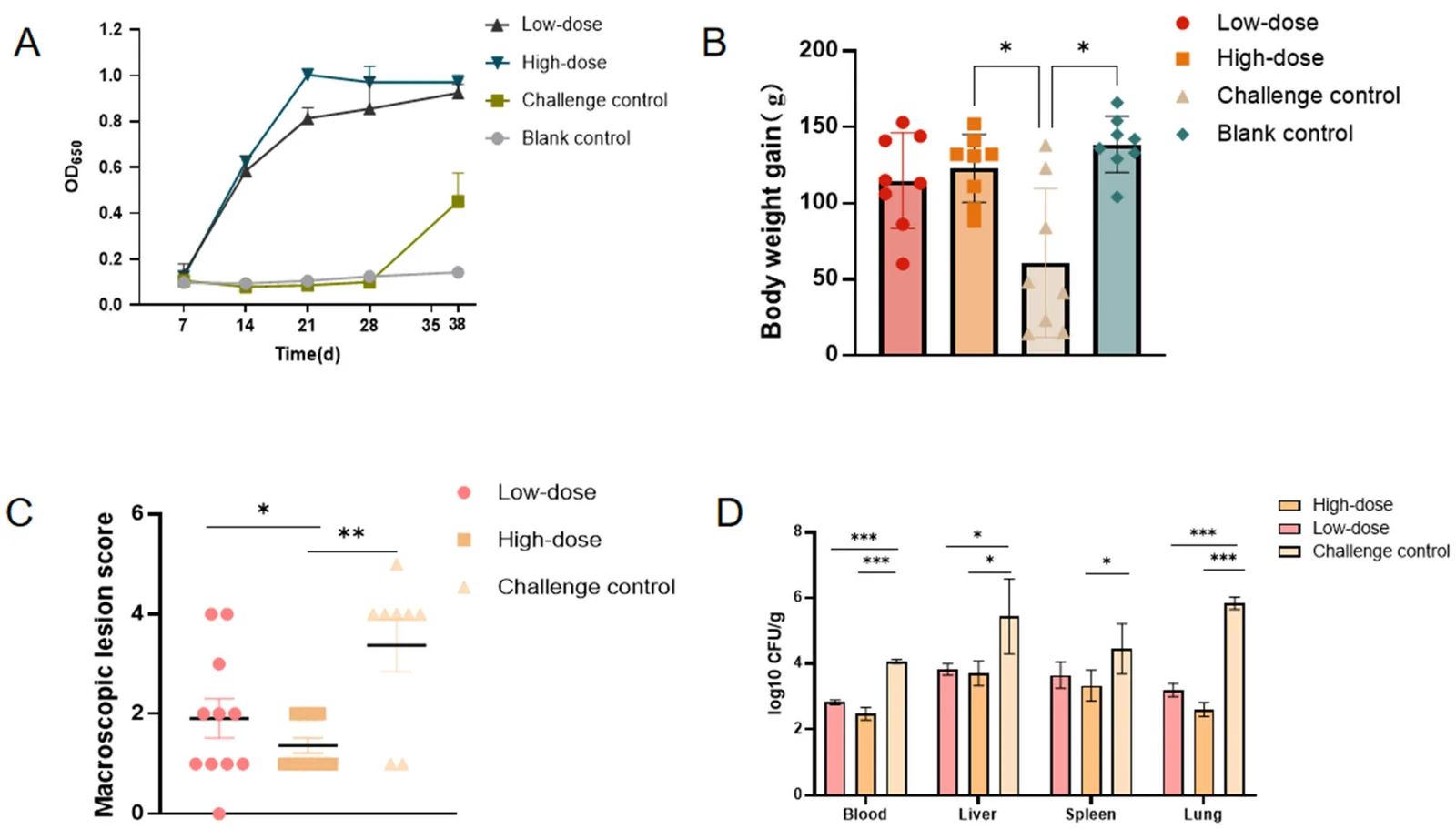
Immunogenicity and protective efficacy of the O18–CRM197 glycoconjugate candidate after homologous APEC O18 challenge. (**A**) O18-specific serum IgG responses induced by O18–CRM197 glycoconjugate immunization. Serum samples were collected every 7 days after immunization and at 10 days post-challenge. Data are presented as mean ± SEM; *n* = 4 chickens per group at each time point. (**B**) Body weight gain before and after homologous APEC O18 challenge. Data are presented as mean ± SD; *n* = 8 chickens per group. (**C**) Gross lesion scores after homologous APEC O18 challenge. Data are presented as mean ± SD; *n* = 8 chickens per group. (**D**) Bacterial burdens in heart blood, liver, spleen, and lung at 24 h post-challenge. Bacterial burden data were log10-transformed before statistical analysis. Data are presented as mean ± SD; *n* = 5 chickens per challenged group. Exact *p* values are shown in the figure. Asterisks indicate statistically significant differences between groups (* *p* < 0.05, ** *p* < 0.01, *** *p* < 0.001).

Both immunized groups developed detectable O18-specific serum IgG responses after vaccination (Figure 4A). At 14 days after the primary immunization and at 7 and 14 days after booster immunization, the 30 μg immunization group showed significantly higher IgG levels than the blank control group (*p* < 0.05). The 50 μg immunization group showed a more pronounced increase in antibody levels at the same time points (*p* < 0.01). At 10 days post-challenge, O18-specific IgG levels in both immunized groups remained significantly higher than those in the challenge control group (*p* < 0.05). No significant difference was observed between the two immunized groups (*p* > 0.05). These results indicate that the adjuvanted purified O18–CRM197 candidate preparation was immunogenic under the tested conditions.

After homologous APEC O18 challenge, clinical signs were mainly observed during the first 5 days post-challenge. Affected chickens showed depression, ruffled feathers, and loose feces. Chickens in the immunized groups mainly developed greenish loose feces, whereas bloody diarrhea was additionally observed in the challenge control group. Clinical signs gradually improved during the later observation period. Representative clinical observations, including loose feces or diarrhea in challenged chickens, are shown in Supplementary Figure S1A,B the 10-day observation period after challenge, mortality was recorded daily. Six of eight chickens in the challenge control group died after APEC O18 challenge, whereas no mortality was observed in either the 30 μg or 50 μg O18–CRM197 immunization groups, corresponding to mortality rates of 75% and 0%, respectively. These results indicated that O18–CRM197 immunization provided protection against lethal APEC O18 challenge.

Body weight gain was recorded before challenge and at 10 days post-challenge. Chickens in the 50 μg immunization group and the blank control group showed significantly higher body weight gain than those in the challenge control group (*p* < 0.05; Figure 4B). In contrast, body weight gain in the 30 μg immunization group was not significantly different from that in the challenge control group (*p* > 0.05), suggesting that the 50 μg dose partially alleviated challenge-associated growth impairment.

Gross lesions were evaluated at 10 days post-challenge. Chickens in the challenge control group showed obvious lesions, including turbid pericardium, fibrinous exudation on the pericardial surface, hepatomegaly, and mild fibrinous exudation on the liver. In contrast, chickens in the immunized groups showed milder lesions and fewer affected birds. Compared with the challenge control group, the mean lesion score was significantly reduced in the 30 μg immunization group (*p* < 0.05) and more markedly reduced in the 50 μg immunization group (*p* < 0.01; Figure 4C). Representative gross appearances of the heart and liver from each group are shown in Supplementary Figure S1C–F. The challenge control group showed more obvious gross lesions, whereas the immunized groups showed comparatively milder lesions, consistent with the quantitative gross lesion scores.

Bacterial burdens were further determined in blood and major organs at 24 h post-challenge. Bacterial loads in the immunized groups were generally lower than those in the challenge control group (Figure 4D). Except for the splenic bacterial burden in the 30 μg immunization group, which showed no significant difference compared with the challenge control group (*p* > 0.05), bacterial burdens in heart blood, liver, and lung were significantly reduced in both immunized groups. In addition, the 50 μg immunization group showed a significant reduction in splenic bacterial burden compared with the challenge control group.

Taken together, these results show that immunization with the adjuvanted purified O18–CRM197 candidate preparation was associated with O18-specific humoral responses and improved disease-associated outcomes after homologous APEC O18 challenge, including reduced gross lesion severity and bacterial burdens. Under the present experimental design, these findings support the preliminary protective potential of the purified candidate preparation, but do not by themselves distinguish the specific contribution of glycan–protein conjugation from the effects of the individual antigenic components or the white oil–Span adjuvant.

## 4. Discussion

In this study, an O18–CRM197 glycoconjugate vaccine candidate was developed using an engineered *E. coli* production host and evaluated in an SPF chicken homologous challenge model. The main findings were as follows: first, the CLM24-derived production host Y03 was successfully constructed by sequential deletion of *lpxM*, *zwf*, and *gldA*, without causing an obvious growth defect under the tested culture conditions; second, the O18 O-polysaccharide module and the modified CRM197-containing module were functionally verified; third, the purified O18–CRM197 preparation showed high-molecular-weight ladder-like signals recognized by both anti-CRM197 antibody and anti-O18 serum, supporting production of an O18–CRM197 glycoconjugate candidate; finally, immunization with this candidate induced O18-specific serum IgG responses and reduced challenge-associated outcomes, including gross lesion severity and bacterial burdens, after homologous APEC O18 challenge. Together, these findings provide preliminary proof-of-concept evidence that a biologically produced O18–CRM197 glycoconjugate strategy can be applied to a poultry APEC O18 challenge model.

APEC remains an important cause of colibacillosis in poultry and is associated with respiratory and systemic infections, economic losses, and increasing concern regarding antimicrobial resistance [16,17]. Because APEC is not represented by a single conserved genotype or serotype, vaccine development remains challenging, and an effective vaccine strategy should consider both antigenic specificity and future serotype coverage. Multiple vaccine approaches have been explored, including live attenuated vaccines, inactivated or autogenous vaccines, recombinant subunit vaccines, outer membrane vesicle-based vaccines, and polysaccharide- or O-antigen-based strategies. Compared to protein-only vaccine strategies, O-antigen-based vaccines have the advantage of directly targeting surface-exposed serotype-specific polysaccharide antigens [18,19,20]. The O-polysaccharide is the distal and highly exposed portion of LPS and is an important target for antibody recognition and complement-related antibacterial activity [21,22]. Therefore, O18 O-polysaccharide was selected as the serotype-specific glycan antigen, while CRM197 was used as the carrier protein to improve the immunogenicity of the polysaccharide component. This design should be interpreted as a serotype-specific vaccine module rather than a single broadly protective APEC vaccine, and it may be more suitable for later incorporation into a multivalent glycoconjugate strategy.

The O-polysaccharide component of lipopolysaccharide is a major surface-exposed antigen and plays an important role in serotype specificity and antibody recognition. Previous studies have shown that O-antigen-based strategies can provide protection against APEC challenge. For example, attenuated Salmonella strains expressing APEC O-antigens have been evaluated as live vaccine vectors and shown to reduce lesions or protect chickens against homologous or related APEC challenge [18]. These studies support the general concept that APEC O-polysaccharides are relevant protective antigens. Compared to live vector-based O-antigen delivery systems, the present strategy focuses on a purified O18–CRM197 candidate preparation, which may offer advantages in terms of defined antigen composition, absence of vaccine-strain replication, and downstream quality control. However, because O-antigen immunity is usually serotype dependent, the protective spectrum of an O18-based candidate needs to be evaluated cautiously and should not be extrapolated to heterologous APEC serotypes without direct challenge data.

Polysaccharide antigens alone are generally considered T cell-independent antigens and may induce weaker or less durable immune responses, particularly in young animals [23]. Coupling polysaccharides to carrier proteins can convert them into T cell-dependent antigens and enhance antibody responses and immunological memory [24]. Mechanistically, glycoconjugate vaccines can be internalized by polysaccharide-specific B cells, after which carrier-derived peptides or glycopeptide-related structures can contribute to T-cell help, germinal center responses, antibody class switching, and memory formation [25,26]. This mechanism is particularly relevant for young chickens, in which responses to unconjugated polysaccharide antigens may be insufficient. CRM197 has been widely used as a carrier protein in licensed human conjugate vaccines and experimental glycoconjugate vaccine platforms because of its safety profile and carrier function. The lack of enzymatic toxicity of CRM197 and its established use as a carrier further support its selection as the protein component in this study [27]. Therefore, the use of CRM197 as the carrier protein in this study is consistent with established conjugate vaccine principles.

PGCT provides a modular biological route for producing glycoconjugates in engineered bacterial cells and has been increasingly explored for vaccine development [28]. In PGCT systems, bacterial oligosaccharyltransferase PglB transfers glycan substrates to acceptor proteins containing a recognizable glycosylation sequon, allowing in vivo biosynthesis of glycoprotein antigens [29,30,31]. Previous studies have demonstrated the feasibility of PGCT-based approaches for producing glycoconjugate vaccine candidates, including multivalent poultry vaccine candidates and *E. coli* O-antigen glycoconjugates [32]. The present study extends this concept to an APEC O18 O-polysaccharide-CRM197 candidate and evaluates it in an SPF chicken homologous challenge model, thereby linking glycoengineering-based production with preliminary immunoprotective evaluation in the target avian host.

Y03 was constructed from the previously established PGCT-compatible strain CLM24, which lacks *waaL*. In PGCT systems, deletion of *waaL* is important because it prevents ligation of O-polysaccharide to lipid A-core and increases the availability of glycan substrates for PglB-mediated transfer to acceptor proteins. In this study, *lpxM*, *zwf*, and *gldA* were further deleted from the CLM24 background as rational engineering steps for constructing the production host. Among these modifications, deletion of *lpxM* is theoretically relevant to lipid A detoxification because *lpxM* participates in late acyl-chain modification of lipid A, and APEC *lpxM* has been associated with pathogenicity in a chicken infection model [33]. In addition, engineering lipid A acylation has been used as a strategy to reduce endotoxic activity in *E. coli* expression hosts [34]. The deletion of *zwf* and *gldA* was intended to redirect carbon metabolism and improve the availability of sugar precursors for polysaccharide synthesis [35,36,37]. Growth curve analysis showed that these deletions did not cause an obvious growth defect under the tested conditions. However, lipid A structure, endotoxin activity, glycosylation efficiency, and production yield were not directly compared between CLM24 and Y03. Therefore, the current data support Y03 as a CLM24-derived engineered production host for O18–CRM197 candidate production, rather than as a fully optimized or safety-validated chassis. Further studies comparing CLM24 and Y03 will be needed to define the functional contribution of these additional genome modifications, particularly in terms of lipid A structure, endotoxin activity, glycosylation efficiency, and glycoconjugate yield [38].

In the present study, the O18 O-polysaccharide expression module was first verified in W3110, where O18-reactive ladder-like signals were detected by anti-O18 serum. The pCRM197-pglB plasmid encoded PglB and a modified CRM197 carrier protein containing a signal peptide, a PglB-recognized glycosylation acceptor sequence, and a His tag. This design provided the necessary components for PglB-mediated glycan transfer and downstream affinity purification. The purified product from Y03/pCRM197-pglB/pET-32a(+)-O18 showed high-molecular-weight ladder-like signals above 100 kDa when detected with anti-CRM197 antibody, and shifted O18-reactive signals when detected with anti-O18 serum. The detection of high-molecular-weight signals by both antibodies is consistent with production of an O18–CRM197 glycoconjugate candidate. Nevertheless, this evidence should be interpreted as supportive rather than definitive structural proof of covalent conjugation. Future confirmation should include mass spectrometry-based glycosylation-site mapping, glycan profiling, glycan-to-protein ratio determination, residual endotoxin testing, and genetic negative controls such as pglB-deficient or glycosylation-sequon-mutated CRM197 controls. These analyses would strengthen the structural and quality-control evidence required for further vaccine development.

The immunization experiment showed that both 30 μg and 50 μg doses induced detectable O18-specific serum IgG responses in SPF chickens. The antibody response became more evident after booster immunization, indicating that the O18–CRM197 glycoconjugate candidate was immunogenic under the tested conditions. The 50 μg group generally showed stronger or more consistent antibody responses than the 30 μg group, although the difference between the two immunized groups was not significant at all time points. These results are consistent with the expected role of glycoconjugate vaccines, in which coupling a polysaccharide antigen to a carrier protein can enhance antigen recognition and promote stronger adaptive immune responses compared with polysaccharide antigen alone. Because only ELISA-binding IgG was measured in this study, future work should determine whether these antibodies have functional antibacterial activity, such as complement-mediated serum bactericidal activity or opsonophagocytic activity [39,40,41].

After homologous APEC O18 challenge, immunized chickens showed improved protective outcomes. The 50 μg immunization group showed significantly higher body weight gain than the challenge control group, suggesting partial alleviation of challenge-associated growth impairment. Gross lesion scores were also reduced in both immunized groups, with the 50 μg group showing a more pronounced reduction. In addition, bacterial burdens in heart blood, liver, lung, and spleen were generally lower in immunized chickens than in challenge controls. The parallel induction of O18-specific IgG and reduction in early bacterial burdens suggests that humoral immunity against surface-exposed O18 O-polysaccharide may have contributed to limiting systemic bacterial dissemination after challenge. The subsequent reduction in gross lesions and preservation of body weight gain, particularly in the 50 μg group, likely reflected decreased bacterial invasion and reduced tissue damage. Overall, the 50 μg dose showed more consistent protective performance than the 30 μg dose in this experimental model. However, because comparator groups containing unconjugated O18 polysaccharide, CRM197 alone, physical mixtures, or adjuvant alone were not included, the specific contribution of glycan-protein conjugation to the observed protection cannot be independently determined in the current study.

Despite these encouraging findings, several limitations should be noted. First, only homologous APEC O18 challenge was evaluated; therefore, whether the O18–CRM197 candidate provides cross-protection against heterologous APEC serotypes remains unclear. Given the antigenic diversity of APEC, we are currently constructing additional APEC serotype-specific glycoconjugate candidates with the aim of expanding serotype coverage and developing a broader multivalent strategy. Second, unconjugated O18 polysaccharide, CRM197 alone, physical mixture, and adjuvant-only control groups were not included. This animal experiment was designed as an initial proof-of-concept and dose-evaluation study to determine whether the adjuvanted purified O18–CRM197 candidate preparation could induce O18-specific immune responses and improve outcomes after homologous challenge. Therefore, the present data demonstrate the preliminary protective potential of the adjuvanted purified candidate preparation, rather than definitive proof that protection was mediated exclusively by covalent glycan–protein conjugation. Third, structural characterization of the glycoconjugate was mainly based on immunoblotting and protein/carbohydrate content determination. Further studies including additional antigen and adjuvant control groups, structural analyses, and broader challenge models are needed to clarify the contribution of glycan conjugation and evaluate wider protective efficacy [42].

## 5. Conclusions

In this study, a CLM24-derived engineered *E. coli* production host was constructed and used to produce a purified O18–CRM197 glycoconjugate candidate preparation. The O18 O-polysaccharide and CRM197-containing expression modules were successfully verified, and the purified preparation showed immunoreactive high-molecular-weight signals recognized by both anti-CRM197 antibody and anti-O18 serum, supporting production of an O18–CRM197 glycoconjugate candidate.

Immunization with the adjuvanted purified O18–CRM197 candidate preparation induced O18-specific serum IgG responses in SPF chickens and was associated with reduced gross lesion severity and bacterial burdens after homologous APEC O18 challenge. The 50 μg dose showed more consistent protective performance than the 30 μg dose under the tested conditions. Overall, these findings provide preliminary proof-of-concept evidence that the purified O18–CRM197 candidate preparation has protective potential against homologous APEC O18 challenge and may support further development of multivalent APEC glycoconjugate vaccine candidates.

## Figures and Tables

**Table 1:** Bacterial strains, plasmids, antibodies, and animals used in this study.

Category	Name	Relevant Information	Source/Reference
Strain	*E. coli* W3110	Original/reference E. coli strain	Laboratory stock
Strain	CLM24	W3110-derived ΔwaaL PGCT-compatible chassis	Laboratory stock
Strain	Y01	CLM24 ΔlpxM	This study
Strain	Y02	CLM24 ΔlpxM Δzwf	This study
Strain	Y03	W3110 ΔwaaL ΔlpxM Δzwf ΔgldA	This study
Strain	*E. coli* DH5α	Plasmid construction and propagation	Laboratory stock
Strain	APEC O18	Source of the O18 O-polysaccharide biosynthesis gene cluster and homologous challenge strain	Laboratory stock
Plasmid	pHCY-25A	Helper plasmid carrying Cas9, the λ-Red recombination system, and the sacB counter-selection marker	Laboratory stock
Plasmid	pHCY-26D-lpxM	Editing plasmid for lpxM deletion	This study
Plasmid	pHCY-26D-zwf	Editing plasmid for zwf deletion	This study
Plasmid	pHCY-26D-gldA	Editing plasmid for gldA deletion	This study
Plasmid	pET-32a(+)	Backbone vector	Laboratory stock
Plasmid	pET-32a(+)-O18	O18 O-polysaccharide expression plasmid	This study
Plasmid	pCRM-4	Original PglB–CRM197 plasmid	Laboratory stock
Plasmid	pCRM197-pglB	PglB and modified CRM197 co-expression plasmid	This study
Antibody	Rabbit anti-O18 serum	Detection of O18 O-polysaccharide	Laboratory stock
Antibody	Anti-CRM197 antibody	Detection of CRM197	GeneTex, Irvine, CA, USA, GTX637460
Antibody	HRP-conjugated goat anti-rabbit IgG	Secondary antibody for Western blotting	Sigma-Aldrich, St. Louis, MO, USA, 12-348
Antibody	HRP-conjugated goat anti-chicken IgG	Secondary antibody for ELISA	Sigma-Aldrich, St. Louis, MO, USA, 12-349
Animal	SPF chickens	Immunization and homologous challenge experiment	Boehringer Ingelheim Vital Biotechnology Co., Ltd., Beijing, China

## Data Availability

The original contributions presented in this study are included in the article/Supplementary Material. Further inquiries can be directed to the corresponding author.

## Supplementary Materials

The following supporting information can be downloaded at: https://www.mdpi.com/article/10.3390/microorganisms14092082/s1, Figure S1: Representative clinical observations and gross lesions after homologous APEC O18 challenge; Table S1: Primers used in this study.

## Abbreviations

The following abbreviations are used in this manuscript:

APEC	avian pathogenic *Escherichia coli*
CRM197	cross-reacting material 197
PGCT	protein glycan coupling technology
SPF	specific-pathogen-free chickens
O-PS	O-polysaccharide
LPS	lipopolysaccharide
CFU	colony-forming units
IgG	immunoglobulin G
HRP	horseradish peroxidase
ELISA	enzyme-linked immunosorbent assay
ARRIVE	Animal Research: Reporting of In Vivo Experiments guidelines
IPTG	isopropyl β-D-1-thiogalactopyranoside
PCR	polymerase chain reaction
LB	Luria–Bertani broth
OD600	optical density at 600 nm
PBS	phosphate-buffered saline
PBST	phosphate-buffered saline containing Tween-20
SDS-PAGE	sodium dodecyl sulfate–polyacrylamide gel electrophoresis
TB	terrific broth medium
Ni-NTA	nickel-nitrilotriacetic acid affinity chromatography
BCA	bicinchoninic acid assay
ANOVA	analysis of variance
