# Supplementary material for: Development and Protective Evaluation of an O18–CRM197 Glycoconjugate Candidate Preparation Against APEC O18 Challenge in SPF Chickens
Source: Microorganisms. 2026 Sep 17;14(9):2082. doi: 10.3390/microorganisms14092082 (PMC13609538; doi:10.3390/microorganisms14092082)
Supplement: Supplementary file 1 [file microorganisms-14-02082-s001.zip › Supplementary Figure S1.pdf]

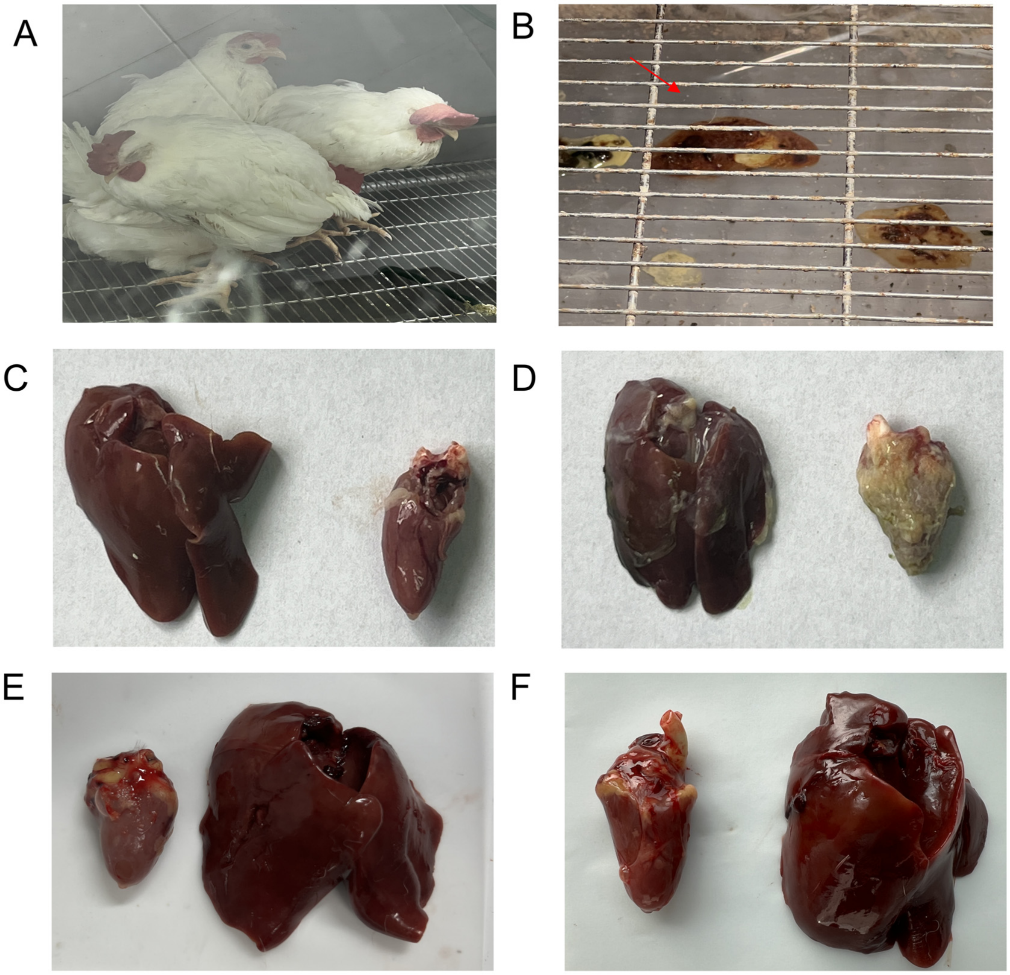

Supplementary Figure S1. Representative clinical observations and gross lesions after homologous APEC O18 challenge.

(A) Representative clinical appearance of chickens after homologous APEC O18 challenge. (B) Representative loose feces or diarrhea observed in challenged chickens, as indicated by the red arrow. (C–F) Representative gross appearance of the heart and liver from the blank control group (C), challenge control group (D), low-dose immunized challenged group receiving 30 μg/bird of the adjuvanted purified O18–CRM197 candidate preparation (E), and high-dose immunized challenged group receiving 50 μg/bird (F). The challenge control group showed more obvious gross lesions, whereas the immunized groups showed comparatively milder lesions.
