# Supplementary material for: Development and Protective Evaluation of an O18–CRM197 Glycoconjugate Candidate Preparation Against APEC O18 Challenge in SPF Chickens
Source: Microorganisms. 2026 Sep 17;14(9):2082. doi: 10.3390/microorganisms14092082 (PMC13609538; doi:10.3390/microorganisms14092082)
Supplement: Supplementary file 1 [file microorganisms-14-02082-s001.zip › Supplementary Table S1.pdf]

Supplementary Table S1. Primers used in this study

Primer name	Sequence (5′–3′)	Target/Use	Expected size	Notes
lpxM-F/R	*CTCGCCTCTGACGTGGTAATG / GCAGTGGCAAATGGATTTCC*	*lpxM* deletion verification	2,617 bp parental / 1,645 bp mutant	PCR verification
zwf-F/R	TAGCAGCGACAATTCCATTCC/ATTCACCGTCGGTTCGCTAA	*zwf* deletion verification	2,584 bp parental / 1,278 bp mutant	PCR verification
gldA-F/R	CGATTGGTGATGAGGGCAT/GTGATCGATGTTGCTGTCCG	*gldA* deletion verification	2,260 bp parental / 1,100 bp mutant	PCR verification
O18-1F/O18-1R	TCGATCAGGTAACTGCTCAGTTC/CCATTCTAATGGCCACCTACAA	O18-1 fragment amplification	5090 bp	O18 O-PS cluster
O18-2F/O18-2R	TTGTAGGTGGCCATTAGAATGG/GCGGAAGTGCAGTTCATCTG	O18-2 fragment amplification	7007 bp	O18 O-PS cluster
F03/F04	CAGATGAACTGCACTTCCGCATGTATATCTCCTTCTTAAAGTTAAACAA/CTGAGCAGTTACCTGATCGAGATCCGGCTGCTAACAAAGC	Linearization of pET-32a(+)	5384 bp	Vector backbone
443/F07	CATACTCTGCGACATCGTATAACGT/GCAGAAAGCACATAACGGCC	pET-32a(+)-O18 colony PCR	915 bp	Plasmid verification
F05/O18-3R	GATTGTACCCTCACCATC/AAGTTTTTGGTGACGAGCGC	W3110/pET-32a(+)-O18 verification	3395 bp	Transformant verification
